# Immunization Coverage and Barriers among Hajj and Umrah Pilgrims: Insights into Vaccine Uptake and Compliance

**DOI:** 10.1007/s44197-025-00447-1

**Published:** 2025-08-07

**Authors:** Adeeb A. Bulkhi, Hani M. Almoallim, Majed S. Obaid, Nahla H. Hariri, Hamsah S. Alqashqri, Ismail A. Alghamdi, Amar Mohammad A. Alkhotani, Muhammad Irfanullah Siddiqui, Heba M. Adly, Mohammed A. Garout, Elbagir A. Elfaki, Saleh A. K. Saleh, Noura Mohammed Bakhsh, Raghda Sami Hassan H. Alhazmi, Aous S. Alhazmi, Jaffar A. Al-Tawfiq, Fahad A. Alamri, Anas A. Khan

**Affiliations:** 1 Internal Medicine Department, College of Medicine, Umm Al-Qura University, Makkah, 21955 Saudi Arabia; 2 Department of Community Medicine and Pilgrims Health care, College of Medicine, Umm Al-Qura University, Makkah, 21955 Saudi Arabia; 3 Surgery Department, King Faisal Hospital, Makkah, Saudi Arabia; 4 Directorate of Institutional Excellence, Batterjee Medical College, Jeddah, 21442 Saudi Arabia; 5 Ajyad Emergency Hospital, Ministry of Health, Makkah, Saudi Arabia; 6 Diabetology and endocrine Centre, Hera’a Hospital, Makkah, Saudi Arabia; 7 Occupational Medicine specialist, Makkah, Saudi Arabia; 8 Specialty Internal Medicine and Quality Department, Johns Hopkins Aramco Healthcare, Dhahran, Saudi Arabia; 9 Infectious Diseases Division, Department of Medicine, Indiana University School of Medicine, Indianapolis, IN USA; 10 Infectious Diseases Division, Department of Medicine, Johns Hopkins University School of Medicine, Baltimore, MD USA; 11 Global Centre for Mass Gatherings Medicine, Ministry of Health, Riyadh, Saudi Arabia; 12 Department of Emergency Medicine, College of Medicine, King Saud University, Riyadh, Saudi Arabia

**Keywords:** Hajj, Vaccine uptake, Mass gatherings, Vaccine barriers, Infectious disease prevention, Public health

## Abstract

**Background:**

The Hajj pilgrimage poses significant public health challenges due to the risk of infectious disease transmission. Despite mandatory vaccination policies, barriers such as hesitancy, logistical issues, and vaccine availability persist.

**Objective:**

This study assesses vaccine uptake, reasons for compliance or non-compliance, and barriers among Hajj pilgrims.

**Methods:**

A cross-sectional study was conducted during the 2024 Hajj season, with data collected via structured interviews from 5,355 participants. Descriptive statistics and logistic regression analyzed vaccination status and influencing factors.

**Results:**

Of the participants, 4298 (80.3%) reported receiving mandatory or recommended vaccines, including influenza, COVID-19, and meningococcal. Primary motivators for vaccination were compliance to mandatory requirements 2240 (52.1%) and healthcare provider recommendations 2018 (46.9%). Among non-vaccinated respondents, the main reasons included belief in vaccine ineffectiveness 250(23.7%), reliance on natural immunity 243(23%), and lack of awareness 174(16.5%). Common barriers included long wait times 721(13.5%) and limited access to vaccination centers 543(10.1%). Women and individuals with higher education demonstrated greater vaccine uptake, while logistical barriers were more prevalent among self-employed and less-educated participants.

**Conclusion:**

Despite high vaccine uptake, barriers remain. Addressing hesitancy, improving access, and aligning vaccine availability with Hajj timing are essential to enhance coverage and reduce disease transmission risks.

**Clinical Trial Number:**

Not applicable.

## Introduction

The Hajj and Umrah pilgrimages are among the world’s largest mass gatherings (MGs), attracting millions of Muslims annually to Makkah, Saudi Arabia. MGs events create unique public health challenges due to overcrowding, physical exertion, environmental factors and the diverse origins of participants, with elevated risk of the spread of infectious diseases, particularly respiratory, gastrointestinal, and vector-borne illnesses [1, 2]. These conditions are taken into consideration by the Saudi Ministry of Health (MoH) to implement rigorous surveillance and preventive measures [1, 3].

In collaboration with the World Health Organization (WHO), the Saudi MoH has established strict health measures for pilgrims to mitigate the risk of infectious disease outbreaks. A list of vaccinations, mainly against meningococcal disease, influenza and COVID-19, is required or recommended to limit the spread of contagious diseases [1, 4]. Surveillance systems such as the Health Early Warning System (HEWS) are continuously updated to address emerging threats [5, 6]. Despite these measures, challenges persist, including the seasonal mismatch of available influenza vaccine due to the lunar Islamic calendar and logistical issues that limit access to vaccinations before Hajj [4, 7].

Non-pharmaceutical interventions, such as hand hygiene and face masks, are also promoted, though their effectiveness is mixed due to inconsistent adherence among pilgrims. On the other hand, social distancing cannot be applied in religious MGs [2, 8].

During the 2024 Hajj season, Saudi health regulations required international pilgrims to present documented evidence of receiving the quadrivalent meningococcal (ACYW) and COVID-19 vaccines prior to visa issuance. Influenza vaccination was also recommended. For Umrah, the meningococcal vaccine remained mandatory for individuals applying through the official Umrah visa route, while COVID-19 and influenza vaccinations were advised. However, a significant number of individuals performing Umrah under tourist or visitor visas were not obligated to submit proof of meningococcal vaccination, although it was strongly encouraged by health authorities. Domestic pilgrims followed the routine national immunization schedule. Additional vaccine requirements were enforced for pilgrims traveling from regions with endemic diseases such as yellow fever or poliomyelitis [9]. Despite these preventive measures, several factors continued to limit vaccine coverage—such as limited awareness, cultural resistance, and operational barriers. Vaccination rates remained suboptimal among local pilgrims, despite their exposure to similar risks [1, 3, 7]. This study included pilgrims from all visa categories.

This study assesses vaccine uptake among Hajj and Umrah pilgrims and the barriers preventing full compliance. The primary objectives are to determine vaccine coverage, explore reasons for receiving or not receiving vaccines, and identify obstacles to vaccination. Understanding these factors is crucial for developing targeted interventions to improve vaccination rates and prevent infectious disease outbreaks during Hajj or Umrah.

## Methodology

### Setting

The study focused on Hajj and Umrah pilgrims visiting Makkah, where official pilgrims reached 1.83 million during the Hajj season mid-2024, and over 26 million throughout 2024 [10]. Pilgrims’ diverse cultural backgrounds influenced vaccine uptake, necessitating a large, representative sample for generalizable results. The 2024 Hajj cohort included participants from multiple countries to ensure diversity. Culturally sensitive questionnaires and rigorous sampling methods were employed to respect participants’ backgrounds while ensuring data accuracy.

### Study Design

This study is a cross-sectional design. Data were collected using structured questionnaires, available in both English and Arabic, designed to gather quantitative information on vaccination rates, reasons for vaccine uptake or refusal, and barriers encountered by the pilgrims. This design facilitated a comprehensive assessment of vaccination patterns among various Hajj attendees. The sampling technique used was cluster sampling followed by a simple random sample.

The inclusion criteria were any pilgrim in Hajj or Umrah between 1 and 30 June. Participants under 18 were excluded. The sample size was calculated using OpenEpi 3.0, accounting for an estimated 2 million pilgrims, a 99% confidence level, 50% frequency, and a design effect of eight to address variability across global clusters. This yielded a minimum of 5,305 participants. To account for non-response and improve reliability, 5,500 participants were recruited. Of them, 5,407 returned the filled forms, and 5,355 were included in the final analysis after excluding 52 incomplete responses.

A structured questionnaire was administered through face-to-face interviews at multiple sites across Makkah, including Al-Haram, Mina, Arafat, Muzdalifah, and surrounding hotels and healthcare facilities. The interviews were conducted by approximately 100 trained data collectors, comprising both male and female junior healthcare professionals such as interns and senior medical students. Gender-matched interviewers were assigned when appropriate to ensure cultural sensitivity.

The questionnaire consisted of around 40 structured questions, addressing demographics, travel and pilgrimage details, general health status, vaccination uptake, motivators and barriers to vaccination, and post-pilgrimage health outcomes. On average, interviews took 10 to 15 min to complete. The tool was available in both Arabic and English. To accommodate pilgrims speaking other languages, interviewers relied on basic multilingual communication and assistance from fellow group members when needed.

Data were recorded using a mix of pen-and-paper forms and electronic tablets, depending on site logistics. The questionnaire was originally developed in Arabic and translated into English using a forward–backward translation method, followed by expert review for conceptual accuracy and cultural appropriateness. A pilot study was conducted to assess clarity and relevance, and subsequent adjustments were made.

All data collectors underwent structured training on standardized administration procedures, ethical conduct, and minimizing interviewer bias. While vaccination status was self-reported by participants, no official documentation, such as immunization cards or visa-related medical records, was requested. Therefore, the responses were based solely on participant recall and awareness of the vaccines received.

### Ethical Part & Confidentiality

After explaining the study goals, verbally informed consent was obtained. Anonymity and confidentiality were ensured, and no personal verifiable data was collected. Data was securely stored and accessible only to researchers.

### Data Analysis

Data were analyzed using descriptive statistics to summarize demographic characteristics and vaccination coverage and identify barriers to vaccine uptake. Chi-square tests were employed to assess associations between key variables, such as demographic factors, vaccine uptake, and barriers faced by the participants. Statistical analyses were performed using IBM SPSS Statistics (Version 26), with all assumptions thoroughly checked to ensure the accuracy and validity of the results.

## Results

Table 1. shows that among the 5,355 participants, the majority were male (3,370; 62.9%), and nearly half held higher education degrees (2,485; 46.4%). Participants represented diverse regions, with the highest proportion from the Eastern Mediterranean Region (2,765; 51.6%), followed by the African Region (972; 18.2%) and the South-East Asia Region (620; 11.6%). A substantial majority (4,603; 86.0%) had not performed Hajj in the past five years. Among those with prior Hajj experience, 382 (61.4%) had attended two to three times, and 74 (11.9%) had completed the pilgrimage more than five times. Most pilgrims traveled by air (4,702; 87.8%). The vast majority (4,364; 81.5%) reported traveling with a group, while 991 (18.5%) traveled individually. Hajj trips were primarily organized through travel agencies (3,432; 64.1%), followed by self-organization (953; 17.8%) and religious organizations (691; 12.9%). A small proportion of participants received support from Tabung Hajj (75; 1.4%) or government/official entities (11; 0.2%), while others listed miscellaneous organizers or left responses incomplete as missing. Self-employment was the most commonly reported occupation (1,440; 27.1%), followed by employment in the private sector (1,207; 22.8%).

**Table 1:** Demographic characteristics of respondents (*N* = 5355)

Demographic Characteristics		*N* (%)
Gender	Male	3370 (62.9%)
	Female	1985 (37.1%)
Residence Region	African Region	972 (18.2%)
	Eastern Mediterranean Region	2764 (51.6%)
	European Region	542 (10.1%)
	Region of the Americas	106 (2.0%)
	South- East Asia Region	620 (11.6%)
	Western Pacific Region	351 6.6%
What is the ritual service you are preforming?	Hajj	4794 (89.5%)
	Umrah	561(10.5%)
Port of Entry	Airport	4702 (87.8%)
	Seaport	32 (0.6%)
	Overland port	621 (11.6%)
Have you participated in Hajj in the past 5 years?	Yes	622 (11.6%)
	No	4603 (86.0%)
	Can’t remember	130 (2.4%)
Did you travel with a group or individually?	Group	4364 (81.5%)
	Individually	991 (18.5%)
Who organized your Hajj trip?	Travel agency	3432 (64.1%)
	Religious organization	691 (12.9%)
	Self-organized	953 (17.8%)
	Tabung Hajj	75 (1.4%)
	Government/Ministry	11(0.2%)
	Nusuk	11 (0.2%)
	Other	86 (1.6%)
	Missing	96 (1.8%)
Education Level	No formal education	658 (12.3%)
	Primary education	624 (11.7%)
	Secondary education	1340 (25.0%)
	Higher education (Bachelor’s degree or equivalent)	2485 (46.4%)
	Postgraduate education (Master’s/PhD)	248 (4.6%)
Occupation (valid = 5304)	Healthcare worker	164 (3.1%)
	Government employee	910 (17.2%)
	Private sector employee	1207 (22.8%)
	Self-employed	1440 (27.1%)
	Unemployed	727 (13.7%)
	Retired	386 (7.3%)
	Student	265 (5.0%)
	Other	105 (2.0%)
	Housewife	100 (1.9)
	Missing	51 (0.9%)

Among the 5,352 surveyed pilgrims, the most commonly received vaccines were the COVID-19 (79.9%) and meningococcal ACWY (79.0%) vaccines, reflecting their mandatory status for Hajj in 2024. However, despite its mandatory nature, meningococcal vaccine uptake was not 100%, which may be attributed to recall bias or a lack of awareness among participants regarding the exact vaccine names recorded on their vaccination cards. Influenza vaccine uptake was lower at 60.2%, consistent with its recommended, but not require status. Uptake of hepatitis A and hepatitis B vaccines was also comparatively low, at 41.7% and 39.5%, respectively. As pilgrims could receive more than one vaccine, the reported percentages are not mutually exclusive and do not sum to 100% (Table 2).

**Table 2:** Vaccination uptake by type of vaccine among pilgrims (*n* = 5352)

Vaccine Type	Received *N* (%)
Meningococcal ACWY	4321 (79.0%)
COVID-19	4116 (79.9%)
Influenza	3220 (60.2%)
Hepatitis A	2230 (41.7%)
Hepatitis B	2112 (39.5%)

* Respondents may have received more than one vaccine; therefore, counts and percentages are not mutually exclusive and do not sum to 100%. Denominators for all vaccine types; meningococcal, COVID-19, influenza, hepatitis A, hepatitis B were (*n* = 5352)

Overall, 4,298 (80.3%) of the respondents reported receiving the recommended vaccines for Hajj and Umrah. These participants demonstrated varying levels of awareness regarding the specific vaccines they had received. When asked to specify the type, 3,007 (56.2%) reported receiving the influenza vaccine, 2,467 (46.1%) indicated COVID-19, and only 1,259 (23.5%) identified the meningococcal (MenACWY) vaccine, despite it being a mandatory requirement for obtaining a Hajj or Umrah visa in 2024. This discrepancy suggests that while the majority complied with mandatory vaccination protocols, there is a notable gap in awareness or recall of the exact vaccine names, particularly concerning the MenACWY vaccine. This highlights the need for improved education and communication regarding vaccine types and their importance.

Among the 1,055 (19.7%) respondents who reported not receiving any vaccines, the most common reasons included belief in vaccine ineffectiveness (250, 23.7%), reliance on natural immunity (243, 23.0%), lack of awareness (174, 16.5%), belief that they rarely become ill (118, 11.2%), and concerns about side effects (105, 10.0%). For those who had received vaccines, the primary motivators were compliance with official Hajj requirements (2,240, 52.1%), recommendations from healthcare providers (2,018, 46.9%), and prevention of illness (1,442, 33.5%) (Table 3).

**Table 3:** Reasons for receiving or not receiving the recommended vaccinations for Hajj or Umrah pilgrimage (*N* = 5355)

Reasons for Not Receiving Recommended Vaccines	*N* (100%)
Believing that the vaccine does not work	250 (23.7%)
Reliance on natural immunity	243 (23)%)
Lack of awareness	174 (16.5)
Believing that they rarely get sick	118 (11.2)
Concern about side effects	105 (10.0%)
Fear of needles	46 (4.4%)
Vaccine unavailability	33 (3.1%)
Personal or family opposition	30 (2.8%)
Cost	18 (1.7%)
Medical contraindications	12 (1.1%)
Other (please specify)	19 (1.8%)
**Reasons for Receiving Recommended Vaccines**	
Requirement by authorities for Hajj participation	2240 (52.1%)
Recommendations from healthcare providers	2018 (46.9%)
To prevent illness	1442 (33.5%)
Personal or family health history	678 (15.8%)
Concern about spreading disease to others	414 (9.6%)
Previous positive experience with vaccinations	404 (9.4%)
Accessibility and convenience of vaccination services	342 (8.0%)
Influence from family or friends	256 (6.0%)
Other	24 (0.6%)

Note: Total sum is more than 1055 since more than one reason given by most respondents

A total of 2284 (42.7%) participants were very aware of the mandatory and recommended Hajj vaccines, while 2185 (40.8%) were somewhat aware, and 886 (16.5%) were not aware. The primary sources of vaccine information were healthcare providers (2825, 52.8%), followed by government sources (1817, 33.9%), Hajj travel agencies (1388, 25.9%), family and friends (1364, 25.5%), social media (1347, 25.2%), media outlets (1228, 22.9%), and others (108, 2.0%).

**Fig. 1:**
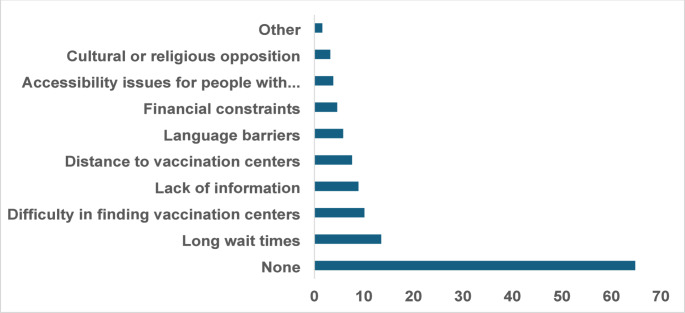
Perceived Challenges in Accessing Vaccines (*N* = 5355)

Figure 1 illustrates the perceived challenges in accessing vaccines among 5355 participants. The most commonly reported challenge was long wait times 721(13.5%), followed by difficulty in finding vaccination centers 543 (10.1%) and lack of information 475 (8.9%). Other challenges included distance to vaccination centers 409 (7.6%), language barriers 313 (5.8%), financial constraints 244 (4.6%), accessibility issues for people with disabilities 204 (3.8%), and cultural or religious opposition 170 (3.2%). However, the majority of participants 3469 (64.8%) reported no challenges.

The most commonly suggested measure to enhance vaccine uptake was providing more information and awareness campaigns, with 2,967 respondents (55.4%) supporting this approach. Easier access to vaccination centers was also highlighted by 2,014 individuals (37.6%), while 1,805 respondents (33.7%) emphasized the importance of educational programs about the benefits of vaccines. Lowering costs or offering vaccines for free was recommended by 1,318 participants (24.6%). Additionally, 1,127 people (21.0%) suggested the use of mobile vaccination units, and 909 respondents (17.0%) believed that support from religious leaders could encourage vaccination. Pre-travel health consultations were mentioned by 773 individuals (14.4%), and 106 respondents (2.0%) proposed other measures.

### Vaccination Uptake and Demographic Variations Among Hajj and Umrah Pilgrims

Vaccine uptake for Hajj was higher among women (83.5%) than men (81.2%, *p* = 0.03), consistent across different age groups. Results revealed significant differences in vaccination uptake across regions. The South-East Asia Region exhibited the highest vaccination uptake at 87.4%, followed closely by the Western Pacific Region (86.3%) and the Eastern Mediterranean Region (83.3%). The African Region recorded the lowest vaccination uptake at 68.2%, indicating potential barriers to vaccination in this population. The Region of the Americas also demonstrated a relatively lower uptake at 49.1%, though the smaller sample size could influence this in this group. Education and occupation also influenced uptake, with higher rates among the higher education (86.3%) and healthcare professionals (86.6%), and lower rates among the uneducated (71.7%) and self-employed (77.1%, *p* < 001) (Table 4).

**Table 4:** Distribution of receiving vaccine related to Hajj by demographic variables

Demographic Characterizes		Total (N)	Status of receiving any vaccines specifically recommended for Hajj or Umrah?		*p*-value*
			Yes	No	
			N (%)	N (%)	
Age Groups (years)	18–39 years	1903	1572 (82.6%)	331 (17.4%)	0.61
	40–59 years	2392	1963 (82.1%)	429 (17.9%)	
	60 + years	1060	860 (81.1%)	200 (18.9%)	
Gender	Male	4395	2737 (81.2%)	633 (18.8%)	0.03
	Female	1985	1658 (83.5%)	327 (16.5%)	
Pilgrimage Residency Region	African Region	972	663 (68.2%)	309 (31.8)	< 0.001
	Eastern Mediterranean Region	2765	2302 (83.3%)	463 (16.7)	
	European Region	541	438 (43.0%)	103 (8.6%)	
	Region of the Americas	106	52 (49.1%)	54 (60.9%)	
	South- East Asia Region	620	542 (87.4%)	78 (12.6%)	
	Western Pacific Region	351	303 (86.3%)	48 (14.7%)	
Education Level	No formal education	658	472 (71.7%)	186 (28.3%)	< 0.001
	Primary education	624	524 (84.0%)	100 (16.0%)	
	Secondary education	1340	1063 (79.3%)	277 (20.7%)	
	Higher education (bachelor’s degree or equivalent)	2485	2145 (86.3%)	340 (13.7%)	
	Postgraduate education (Master’s/PhD)	248	191 (77.0%)	57 (23.0%)	
Occupation	Healthcare worker	164	142 (86.6%)	22 (13.4%)	< 0.001
	Government employee	910	767 (84.3%)	143 (15.7%)	
	Private sector employee	1207	1006 (83.3%)	201 (16.7%)	
	Self-employed	1440	1110 (77.1%)	330 (22.9%)	
	Unemployed	727	620 (85.3%)	107 (14.7%)	
	Retired	386	335 (86.8%)	51(13.2%)	
	Student	265	208 (78.5%)	57 (21.5%)	
	Other	105	91 (86.7%)	14 (13.3%)	
	Housewife	100	85 (85.0%)	15 (15.0%)	

* *p*-values are based on chi-square tests comparing the distribution of vaccine uptake (Yes vs. No) across all categories of each demographic variable (e.g., across age groups, education levels, occupations

### Vaccination Motivations Across Demographics

Younger participants (18–39 years) were primarily influenced by their healthcare provider recommendations (42.8%, *p* < 0.001), while older adults (60 + years) relied more on personal or family health history (20.4%, *p* < 0.001). Prevention of illness was strongest among the oldest age group (33.1%, *p* < 0.001) (Table 5).

**Table 5:** Reasons for Vaccine Uptake by Demographic Variables. Reasons for vaccine uptake by: age groups N (%)

Reason		18–39 years	40–59 years	60 + years	*p*-value*
Recommendations from healthcare providers	Yes	814 (42.8%)	846 (35.4%)	414 (39.1%)	< 0.001
	No	1089 (57.2%)	1546 (64.6%)	646 (60.9%)	
Personal or family health history	Yes	258 (13.6%)	228 (9.5%)	216 (20.4%)	< 0.001
	No	1645 (86.4%)	2164 (90.5%)	844 (79.6%)	
To Prevent illness	Yes	489 (25.7%)	632 (26.4%)	351 (33.1%)	< 0.001
	No	1414 (74.3%)	1760 (73.6%)	709 (66.9%)	
Requirement by authorities for Hajj participation	Yes	633 (33.3%)	1103 (46.1%)	530 (50.0%)	< 0.001
	No	1270 (66.7%)	1289 (53.9%(	530) 50.0%(	
Previous positive experience with vaccinations	Yes	138 (7.3%)	189 (7.9%)	88 (8.3%)	0.55
	No	1765 (92.7%)	2203 (92.1%)	972 (91.7%)	
Concern about spreading disease to others	Yes	156 (8.2%)	175 (7.3%)	92 (8.7%)	0.33
	No	1747 (91.8%)	2217 (92.7%)	968 (91.3%)	
Influence from family or friends	Yes	91(4.8%)	104 (4.3%)	67 (6.3%)	0.045
	No	1812 (95.2%)	2288 (95.7%)	993 (93.7%)	
Accessibility and convenience of vaccination services	Yes	109 (5.7%)	187 (7.8%)	52 (4.9%)	0.001
	No	1794 (94.3%)	2205 (92.2%)	1008 (95.1%)	
Other	Yes	9 (0.5%)	8 (0.3%)	11(1.0%)	0.03
	No	1894 (99.5%)	2384 (99.7%)	1049 (99.0%)	

* *P*-values are derived from chi-square tests comparing the distribution of responses (Yes/No) for each reason across all three age categories (18–39, 40–59, and 60 + years)

In the study, differences emerged among males and females. Males cited their healthcare provider recommendations more often than females (40.4% vs. 36.0%, *p* = 0.001), and personal or family health history more frequently among males than females (14.2% vs. 11.3%, *p* = 0.003). Hajj requirements were a stronger motivator for males (46.6%) than females (46.6% vs. 39.8%, *p* < 0.001) (Table 6).

**Table 6:** Reasons for vaccine uptake by: gender N (%)

Reason		Male	Female	*p*-value*
Recommendations from healthcare providers	Yes	1360 (40.4%)	714 (36.0%)	0.001
	No	2010 (59.6%)	1271(64.0%)	
Personal or family health history	Yes	477 (14.2%)	225 (11.3%)	0.003
	No	2893 (85.8%)	1760 (88.7%)	
To Prevent illness	Yes	919 (27.3%)	553 (27.9%)	0.64
	No	2451 (72.7%)	1432 (72.1%)	
Requirement by authorities for Hajj participation	Yes	1340 (39.8%)	926 (46.6%)	< 0.001
	No	2030 (60.2%)	1059 (53.4%)	
Previous positive experience with vaccinations	Yes	247 (7.3%)	168 (8.5%)	0.13
	No	3123 (92.7%)	1817 (91.5%)	
Concern about spreading disease to others	Yes	271 (8.0%)	152 (7.7%)	0.61
	No	3099 (92.0%)	1833 (92.3%)	
Influence from family or friends	Yes	157 (4.7%)	105 (5.3%)	0.30
	No	3213 (95.3%)	1880 (94.7%)	
Accessibility and convenience of vaccination services	Yes	177 (5.3%)	171(8.6%)	< 0.001
	No	3193 (94.7%)	1814 (91.4%)	
Other	Yes	14 (0.4%)	14 (0.7%)	0.16
	No	3356 (99.6%)	1971(99.3%)	

*P-values indicate the results of Chi-square tests comparing proportions of male and female respondents selecting each reason for vaccine uptake. Each *p*-value assesses whether the difference in response distribution between genders is statistically significant for that specific reason

Education level strongly correlated with healthcare recommendations (*p* < 0.001). Specifically, 45.9% of participants with a bachelor’s degree and 33.1% with postgraduate education reported healthcare advice as the primary motivator. Prevention of illness was prevalent among individuals with secondary education (26.9%) and higher education (32.3%) (Table 7).

**Table 7:** Reasons for vaccine uptake by: education level N (%)

Reason		No formal Education	Primary Education	Secondary Education	Higher education (Bachelor’s degree or equivalent)	Postgraduate education Master’s/PhD	*p*-value*
Recommendations from healthcare providers	Yes	151(22.9%	219 (35.1%)	482 (36.0%)	1140 (45.9%)	82 (33.1%)	< 0.001
	No	507 (77.1%)	405 (64.9%)	858 (64.0%)	1345 (54.1%)	166 (66.9%)	
Personal or family health history	Yes	42 (6.4%)	69 (11.1%)	144 (10.7%)	430 (17.3%)	17 (6.9%)	< 0.001
	No	616 (93.6%)	555 (88.9%)	1196 (89.3%)	2055 (82.7%)	231 (93.1%)	
To Prevent illness	Yes	126 (19.1%)	118 (18.9%)	361 (26.9%)	803 (32.3%)	64 (25.8%)	< 0.001
	No	532 (80.9%)	506 (81.1%)	979 (73.1%)	1682 (67.7%)	184 (74.2%)	
Requirement by authorities for Hajj participation	Yes	273 (41.5%)	280 (44.9%)	538 (40.1%)	1060 (42.7%)	115 (46.4%)	0.18
	No	385 (58.5%)	344 (55.1%)	802 (59.9%)	1425 (57.3%)	133 (53.6%)	
Previous positive experience with vaccinations	Yes	19 (2.9%)	40 ) 6.4%)	95 (7.1%)	237 (9.5%)	24 (9.7%)	< 0.001
	No	639 (97.1%)	584 (93.6%)	1245 (92.9%)	2248 (90.5%)	224 (90.3%)	
Concern about spreading disease to others	Yes	40 (6.1%)	23 (3.7%)	94 (7.0%)	237 (9.5%)	29 (11.7%)	< 0.001
	No	618 (93.9%)	601 (96.3%)	1246 (93.0%)	2248 (90.5%)	219 (88.3%)	
Influence from family or friends	Yes	24 (3.6%)	22 (3.5%(	72 (5.4%)	135 (5.4%)	9 (3.6%)	0.10
	No	634 (96.4%)	602 (96.5%)	1268 (94.6%)	2350 (94.6%)	239 (96.4%)	
Accessibility and convenience of vaccination services	Yes	8 (1.2%)	118 (18.9%)	103 (7.7%)	107 (4.3%)	12 (4.8%)	< 0.001
	No	650 (98.8%)	506 (81.1%)	1237 (92.3%)	2378 (95.7%)	236 (95.2%)	
Other	Yes	4 (0.6%)	9 (1.4%)	2 (0.1%)	13 (0.5%)	0 (0.0%)	0.004
	No	654 (99.4%)	615 (98.6%)	1338 (99.9%)	2472 (99.5%)	248 (100.0%)	

*P-values represent results from Chi-square tests comparing the proportions of participants across different education levels who selected each reason for vaccine uptake. Each *p*-value indicates whether there is a statistically significant association between education level and the given reason

Government employees and retired individuals were more likely to cite Hajj requirements as a primary motivator than self-employed individuals (49.7% vs. 48.7% vs. 33.1%, *p* < 0.001). Healthcare professionals were more influenced by previous positive vaccination experiences than other occupational groups (*p* < 0.001) (Table 8).

**Table 8:** Reasons for vaccine uptake by occupation

Reasons		Healthcare worker	Government Employment	Private sector employee	Self-employed	Unemployed	Retired	Student	Other	Housewife	*p*-value
Recommendations from healthcare providers	Yes	77	365	543	515	251	162	91	28	27	< 0.001
		47.0%	40.1%	45.0%	35.8%	34.5%	42.0%	34.3%	26.7%	27.0%	
	No	87	545	664	925	476	224	174	77	73	
		53.0%	59.9%	55.0%	64.2%	65.5%	58.0%	65.7%	73.3%	73.0%	
Personal or family health history	Yes	27	166	162	135	77	101	24	5	4	< 0.001
		16.5%	18.2%	13.4%	9.4%	10.6%	26.2%	9.1%	4.8%	4.0%	
	No	137	744	1045	1305	650	285	241	100	96	
		83.5%	81.8%	86.6%	90.6%	89.4%	73.8%	90.9%	95.2%	96.0%	
To Prevent illness	Yes	46	317	358	322	152	146	82	14	29	< 0.001
		28.0%	34.8%	29.7%	22.4%	20.9%	37.8%	30.9%	13.3%	29.0%	
	No	118	593	849	1118	575	240	183	91	71	
		72.0%	65.2%	70.3%	77.6%	79.1%	62.2%	69.1%	86.7%	71.0%	
Requirement by authorities for Hajj participation	Yes	83	452	476	476	335	188	94	73	69	< 0.001
		50.6%	49.7%	39.4%	33.1%	46.1%	48.7%	35.5%	69.5%	69.0%	
	No	81	458	731	964	392	198	171	32	31	
		49.4%	50.3%	60.6%	66.9%	53.9%	51.3%	64.5%	30.5%	31.0%	
Previous positive experience with vaccinations	Yes	35	83	80	95	35	53	19	5	8	< 0.001
		21.3%	9.1%	6.6%	6.6%	4.8%	13.7%	7.2%	4.8%	8.0%	
	No	129	827	1127	1345	692	333	246	100	92	
		78.7%	90.9%	93.4%	93.4%	95.2%	86.3%	92.8%	95.2%	92.0%	
Concern about spreading disease to others	Yes	27	97	82	95	45	44	14	5	14	< 0.001
		16.5%	10.7%	6.8%	6.6%	6.2%	11.4%	5.3%	4.8%	14.0%	
	No	137	813	1125	1345	682	342	251	100	86	
		83.5%	89.3%	93.2%	93.4%	93.8%	88.6%	94.7%	95.2%	86.0%	
Influence from family or friends	Yes	8	42	43	61	39	36	21	3	9	< 0.001
		4.9%	4.6%	3.6%	4.2%	5.4%	9.3%	7.9%	2.9%	9.0%	
	No	156	868	1164	1379	688	350	244	102	91	
		95.1%	95.4%	96.4%	95.8%	94.6%	90.7%	92.1%	97.1%	91.0%	
Accessibility and convenience of vaccination services	Yes	16	40	48	189	16	20	12	2	5	< 0.001
		9.8%	4.4%	4.0%	13.1%	2.2%	5.2%	4.5%	1.9%	5.0%	
	No	148	870	1159	1251	711	366	253	103	95	
		90.2%	95.6%	96.0%	86.9%	97.8%	94.8%	95.5%	98.1%	95.0%	
Other	Yes	1	4	5	5	4	5	1	2	1	0.26
		0.6%	0.4%	0.4%	0.3%	0.6%	1.3%	0.4%	1.9%	1.0%	
	No	163	906	1202	1435	723	381	264	103	99	
		99.4%	99.6%	99.6%	99.7%	99.4%	98.7%	99.6%	98.1%	99.0%	

*P-values represent Chi-square tests assessing the association between each reason for vaccine uptake and occupational category

### Reasons for Non-Vaccination

Belief in vaccine ineffectiveness was most prevalent among older participants (60 + years) compared to those aged 40–59 and younger groups (18–39 years) (8.8% vs. 6.8% vs. 2.6%, *p* < 0.001). Concerns about side effects were more common in the 40–59 group than younger and older groups (4.9% vs. 3.0% vs. 3.8%, *p* = 0.007). Fear of needles was primarily reported by younger participants (2.2%, *p* = 0.03) (Table 9).

**Table 9:** Reasons for not getting Vaccinated by Demographic Characters. Reasons for not getting vaccines by: age groups N (%)

Reason		18–39 years	40–59 years	60 + years	*p*-value*
Lack of awareness	Yes	104 (5.5%)	131 (5.5%)	50 (4.7%)	0.62
	No	1799 (94.5%)	2261 (94.5%)	1010 (95.3%)	
Reliance on natural immunity	Yes	112 (5.9%)	172 (7.2%)	73 (6.9%)	0.22
	No	1791 (94.1%)	2220 (92.8%)	987 (93.1%)	
Believing that they rarely get sick	Yes	57 (3.0%)	86 (3.6%)	44 (4.2%)	0.24
	No	1846 (97.0%)	2306 (96.4%)	1016 (95.8%)	
Concern about side effects	Yes	58 (3.0%)	118 (4.9%)	40 (3.8%)	0.007
	No	1845 (97.0%)	2274 (95.1%)	1020 (96.2%)	
Medical contraindications	Yes	16 (0.8%)	11(0.5%)	2 (0.2%)	0.052
	No	1887 (99.2%)	2381(99.5%)	1058 (99.8%)	
Believing that the vaccine does not work	Yes	50 (2.6%)	163 (6.8%)	93 (8.8%)	< 0.001
	No	1853 (97.4%)	2229 (93.2%)	967 (91.2%)	
Vaccine unavailability	Yes	27 (1.4%)	33 (1.4%)	11 (1.0%(	0.65
	No	1876 (98.6%)	2359 (98.6%)	1049 (99.0%)	
Cost	Yes	22 (1.2%)	25 (1.0%)	5 (0.5%)	0.17
	No	1881 (98.8%)	2367 (99.0%)	1055 (99.5%)	
Fear of needles	Yes	41(2.2%)	38 (1.6%)	9 (0.8%)	0.03
	No	1862 (97.8%)	2354 (98.4%)	1051 (99.2%)	
Personal or family opposition	Yes	22 (1.2%)	25 (1.0%)	11 (1.0%)	0.93
	No	1881 (98.8%)	2367 (99.0%)	1049 (99.0%)	
Others	Yes	13 (0.7%)	26 (1.1%)	19 (1.8%)	0.02
	No	1890 (99.3%)	2366 (98.9%)	1041 (98.2%)	

* *P*-values represent Chi-square tests assessing the association between each reported reason for not receiving vaccines and age group categories

Males were more likely to believe vaccines were ineffective than females (6.5% vs. 4.3%, *p* = 0.001), while fear of needles was higher among females (2.2% vs. 1.3%, *p* = 0.01) (Table 10). Lack of awareness was most common among participants with no formal education and primary education compared to postgraduate-educated individuals (7.4% vs. 7.2% vs. 2.8%, *p* = 0.003). Belief in vaccine ineffectiveness was also highest among those with no formal education (14.0%, *p* < 0.001) (Table 11).

**Table 10:** Reasons for not getting vaccines by gender N (%)

Reason		Male	Female	*p*-value*
Lack of awareness	Yes	168 (5.0%)	117 (5.9%)	0.15
	No	3202 (95.0%)	1868 (94.1%)	
Reliance on natural immunity	Yes	228 (6.8%)	129 (6.5%)	0.71
	No	3142 (93.2%)	1856 (93.5%)	
Believing that they rarely get sick	Yes	125 (3.7%)	62 (3.1%)	0.26
	No	3245 (96.3%)	1923 (96.9%)	
Concern about side effects	Yes	135 (4.0%)	81(4.1%)	0.89
	No	3235 (96.0%)	1904 (95.9%)	
Medical contraindications	Yes	17 (0.5%)	12 (0.6%)	0.63
	No	3353 (99.5%)	1973 (99.4%)	
Believing that the vaccine does not work	Yes	220 (6.5%)	86 (4.3%)	0.001
	No	3150 (93.5%)	1899 (95.7%)	
Vaccine unavailability	Yes	48 (1.4%)	23 (1.2%)	0.41
	No	3322 (98.6%)	1962 (98.8%)	
Cost	Yes	32 (0.9%)	20 (1.0%)	0.83
	No	3338 (99.1%)	1965 (99.0%)	
Fear of needles	Yes	44 (1.3%)	44 (2.2%)	0.01
	No	3326 (98.7%)	1941(97.8%)	
Personal or family opposition	Yes	32 (0.9%)	26 (1.3%)	0.22
	No	3338 (99.1%)	1959 (98.7%)	
Others	Yes	33 (1.0%)	25 (1.3%)	0.34
	No	3337 (99.0%)	1960 (98.7%)	

* *P*-values represent Chi-square tests comparing the distribution of each reason for not receiving vaccines between male and female respondents

**Table 11:** Reasons for not getting vaccines by: education level N (%)

Reason		No formal Education	Primary Education	Secondary Education	Higher education (Bachelor’s degree or equivalent)	Postgraduate education (Master’s/PhD)	*p*-value*
Lack of awareness	Yes	49 (7.4%)	45 (7.2%)	70 (5.2%)	114 (4.6%)	7 (2.8%)	0.003
	No	609 (92.6%)	579 (92.8%)	1270 (94.8%)	2371 (95.4%)	241 (97.2%)	
Reliance on natural immunity	Yes	63 (9.6%)	57 (9.1%)	80 (6.0%)	137 (5.5%)	20 (8.1%)	< 0.001
	No	595 (90.4%)	567 (90.9%)	1260 (94.0%)	2348 (94.5%)	228 (91.9%)	
Believing that they rarely get sick	Yes	37 (5.6%)	25 (4.0%)	40 (3.0%)	77 (3.1%)	8 (3.2%)	0.02
	No	621 (94.4%)	599 (96.0%)	1300 (97.0%)	2408 (96.9%)	240 (96.8%)	
Concern about side effects	Yes	34 (5.2%)	41 (6.6%)	53 (4.0%)	82 (3.3%)	6 (2.4%)	0.001
	No	624 (94.8%)	583 (93.4%)	1287 (96.0%)	2403 (96.7%)	242 (97.6%)	
Medical contraindications	Yes	3 (0.5%)	3 (0.5%)	6 (0.4%)	16 (0.6%)	1 (0.4%)	0.92
	No	655 (99.5%)	621 (99.5%)	1334 (99.6%)	2469 (99.4%)	247 (99.6%)	
Believing that the vaccine does not work	Yes	92 (14.0%)	37 (5.9%)	63 (4.7%)	93 (3.7%)	21 (8.5%)	< 0.001
	No	566 (86.0%)	587 (94.1%)	1277 (95.3%)	2392 (96.3%)	227 (91.5%)	
Vaccine unavailability	Yes	10 (1.5%)	9 (1.4%)	20 (1.5%)	30 (1.2%)	2 (0.8%)	0.86
	No	648 (98.5%)	615 (98.6%)	1320 (98.5%)	2455 (98.8%)	246 (99.2%)	
Cost	Yes	3 (0.5%)	8 (1.3%)	9 (0.7%)	30 (1.2%)	2 (0.8%)	0.27
	No	655 (99.5%)	616 (98.7%)	1331 (99.3%)	2455 (98.8%)	246 (99.2%)	
Fear of needles	Yes	10 (1.5%)	14 (2.2%)	24 (1.8%)	40 (1.6%)	0 (0.0%)	0.21
	No	648 (98.5%)	610 (97.8%)	1316 (98.2%)	2445 (98.4%)	248 (100.0%)	
Personal or family opposition	Yes	8 (1.2%)	7 (1.1%)	15 (1.1%)	28 (1.1%)	0 (0.0%)	0.58
	No	650 (98.8%)	617 (98.9%)	1325 (98.9%)	2457 (98.9%)	248 (100.0%)	
Others	Yes	6 (0.9%)	18 (2.9%)	16 (1.2%)	16 (0.6%)	2 (0.8%)	< 0.001
	No	652 (99.1%)	606 (97.1%)	1324 (98.8%)	2469 (99.4%)	246 (99.2%)	

* *P*-values represent Chi-square tests comparing the proportion of respondents citing each reason for non-vaccination across different levels of education

Self-employed individuals (11.1%) were most likely to believe in vaccine ineffectiveness compared to government employees and healthcare professionals (11.1% vs. 2.6% vs. 1.2%, *p* < 0.001). Students and self-employed individuals were more likely to rely on natural immunity (9.1% vs. 8.1%, *p* = 0.004) (Table 12).

**Table 12:** Reasons for not getting vaccines by: occupation

Reasons		Healthcare worker	Government Employment	Private sector employee	Self-employed	Unemployed	Retired	Student	Other	Housewife	*p*-value*
Lack of awareness	Yes	5	50	44	80	55	18	15	7	4	0.03
		3.0%	5.5%	3.6%	5.6%	7.6%	4.7%	5.7%	6.7%	4.0%	
	No	159	860	1163	1360	672	368	250	98	96	
		97.0%	94.5%	96.4%	94.4%	92.4%	95.3%	94.3%	93.3%	96.0%	
Reliance on natural immunity	Yes	4	54	85	116	39	15	24	9	3	0.004
		2.4%	5.9%	7.0%	8.1%	5.4%	3.9%	9.1%	8.6%	3.0%	
	No	160	856	1122	1324	688	371	241	96	97	
		97.6%	94.1%	93.0%	91.9%	94.6%	96.1%	90.9%	91.4%	97.0%	
Believing that they rarely get sick	Yes	3	24	32	72	18	11	12	7	4	0.004
		1.8%	2.6%	2.7%	5.0%	2.5%	2.8%	4.5%	6.7%	4.0%	
	No	161	886	1175	1368	709	375	253	98	96	
		98.2%	97.4%	97.3%	95.0%	97.5%	97.2%	95.5%	93.3%	96.0%	
Concern about side effects	Yes	2	23	46	68	41	21	10	2	3	0.02
		1.2%	2.5%	3.8%	4.7%	5.6%	5.4%	3.8%	1.9%	3.0%	
	No	162	887	1161	1372	686	365	255	103	97	
		98.8%	97.5%	96.2%	95.3%	94.4%	94.6%	96.2%	98.1%	97.0%	
Medical contraindications	Yes	0	3	7	7	3	4	4	0	0	0.29
		0.0%	0.3%	0.6%	0.5%	0.4%	1.0%	1.5%	0.0%	0.0%	
	No	164	907	1200	1433	724	382	261	105	100	
		100.0%	99.7%	99.4%	99.5%	99.6%	99.0%	98.5%	100.0%	100.0%	
Believing that the vaccine does not work	Yes	2	24	73	160	22	11	7	3	2	< 0.001
		1.2%	2.6%	6.0%	11.1%	3.0%	2.8%	2.6%	2.9%	2.0%	
	No	162	886	1134	1280	705	375	258	102	98	
		98.8%	97.4%	94.0%	88.9%	97.0%	97.2%	97.4%	97.1%	98.0%	
Vaccine unavailability	Yes	1	10	15	25	7	6	4	1	1	0.85
		0.6%	1.1%	1.2%	1.7%	1.0%	1.6%	1.5%	1.0%	1.0%	
	No	163	900	1192	1415	720	380	261	104	99	
		99.4%	98.9%	98.8%	98.3%	99.0%	98.4%	98.5%	99.0%	99.0%	
Cost	Yes	1	9	12	11	6	6	3	3	0	0.49
		0.6%	1.0%	1.0%	0.8%	0.8%	1.6%	1.1%	2.9%	0.0%	
	No	163	901	1195	1429	721	380	262	102	100	
		99.4%	99.0%	99.0%	99.2%	99.2%	98.4%	98.9%	97.1%	100.0%	
Fear of needles	Yes	1	16	14	22	18	9	5	1	2	0.4
		0.6%	1.8%	1.2%	1.5%	2.5%	2.3%	1.9%	1.0%	2.0%	
	No	163	894	1193	1418	709	377	260	104	98	
		99.4%	98.2%	98.8%	98.5%	97.5%	97.7%	98.1%	99.0%	98.0%	
Personal or family opposition	Yes	1	8	3	21	8	9	7	1	0	0.003
		0.6%	0.9%	0.2%	1.5%	1.1%	2.3%	2.6%	1.0%	0.0%	
	No	163	902	1204	1419	719	377	258	104	100	
		99.4%	99.1%	99.8%	98.5%	98.9%	97.7%	97.4%	99.0%	100.0%	
Others	Yes	2	8	10	7	18	7	1	2	1	0.003
		1.2%	0.9%	0.8%	0.5%	2.5%	1.8%	0.4%	1.9%	1.0%	
	No	162	902	1197	1433	709	379	264	103	99	
		98.8%	99.1%	99.2%	99.5%	97.5%	98.2%	99.6%	98.1%	99.0%	

* *P*-values reflect Chi-square tests comparing the distribution of each reported reason for not receiving vaccination across different occupational groups

### Vaccination Uptake Barriers

Older adults (60 + years) reported the least vaccination barriers, with 68% reporting none (*p* = 0.01). Long waiting times were more common in the 40–59 age group (14.4%, *p* = 0.04), while accessibility issues were primarily among older adults (4.5%, *p* = 0.03) (Table 13).

**Table 13:** Distribution of barriers to vaccine with demographic characteristics. Distribution of barriers to vaccine by: Age Groups

Reason		18–39 years	40–59 years		60 + years		*p*-value		
Difficulty in finding vaccination centers	Yes	211 (11.1%)		245 (10.2%)		87 (8.2%)		0.04	
	No	1692 (88.9%)		2147 (89.8%)		973 (91.8%)			
Long wait times	Yes	257 (13.5%)		345 (14.4%)		119 (11.2%)		0.04	
	No	1646 (86.5%)		2047 (85.6%)		941 (88.8%)			
Lack of Information	Yes	178 (9.4%)		217 (9.1%)		80 (7.5%)		0.23	
	No	1725 (90.6%)		2175 (90.9%)		980 (92.5%)			
Language Barriers	Yes	117 (6.1%)		150 (6.3%)		46 (4.3%)		0.06	
	No	1786 (93.9%)		2242 (93.7%)		1014 (95.7%)			
Financial constraints	Yes	79 (4.2%)		112 (4.7%)		53 (5.0%)		0.53	
	No	1824 (95.8%)		2280 (95.3%)		1007 (95.0%)			
Cultural or religious opposition	Yes	50 (2.6%)		85 (3.6%)		35 (3.3%)		0.22	
	No	1853 (97.4%)		2307 (96.4%)		1025 (96.7%)			
Distance to vaccination centers	Yes	133 (7.0%)		203 (8.5%)		73 (6.9%)		0.11	
	No	1770 (93.0%)		2189 (91.5%)		987 (93.1%)			
Accessibility issues for people with disabilities	Yes	55 (2.9%)		101 (4.2%)		48 (4.5%)		0.03	
	No	1848 (97.1%)		2291 (95.8%)		1012 (95.5%)			
Other	Yes	16 (0.8%)		46 (1.9%)		23 (2.2%)		0.004	
	No	1887 (99.2%)		2346 (98.1%)		1037 (97.8%)			

* *P*-values indicate results of Chi-square tests assessing differences in reported vaccination barriers across age groups for each listed factor

Gender differences showed that men reported fewer barriers than women (66.7% vs. 61.5%, *p* < 0.001). However, women cited long waiting times and distance to centers more often than men (15.0% vs. 8.8%, *p* = 0.02) (Table 14). Postgraduate degree holders were most likely to report no barriers (75%), while primary education holders faced challenges like long wait times (22.3%) and language barriers (14.6%, *p* < 0.001) (Table 6.3).

**Table 14:** Distribution of barriers to vaccine by: gender

Reason		Male	Female	*p*-value*
Difficulty in finding vaccination centers	Yes	323 (9.6%)	220 (11.1%)	0.08
	No	3047 (90.4%)	1765 (88.9%)	
Long wait times	Yes	423 (12.6%)	298 (15.0%)	0.01
	No	2947 (87.4%)	1687 (85.0%)	
Lack of Information	Yes	284 (8.4%)	191 (9.6%)	0.14
	No	3086 (91.6%)	1794 (90.4%)	
Language Barriers	Yes	181 (5.4%)	132 (6.6%)	0.054
	No	3189 (94.6%)	1853 (93.4%)	
Financial constraints	Yes	143 (4.2%)	101 (5.1%)	0.15
	No	3227 (95.8%)	1884 (94.9%)	
Cultural or religious opposition	Yes	99 (2.9%)	71 (3.6%)	0.20
	No	3271 (97.1%)	1914 (96.4%)	
Distance to vaccination centers	Yes	235 (7.0%)	174 (8.8%)	0.02
	No	3135 (93.0%)	1811 (91.2%)	
Accessibility issues for people with disabilities	Yes	103 (3.1%)	101 (5.1%)	< 0.001
	No	3267 (96.9%)	1884 (94.9%)	
Other	Yes	53 (1.6%)	32 (1.6%)	0.91
	No	3317 (98.4%)	1953 (98.4%)	

* *P*-values derived from Chi-square tests comparing reported barriers to vaccination between male and female participants

**Table 15:** Distribution of barriers to vaccine by: education level

Reason		No formal Education	Primary Education	Secondary Education	Higher education (Bachelor’s degree or equivalent)	Postgraduate education (Master’s/PhD)	*p*-value*
Difficulty in finding vaccination centers	Yes	65 (9.9%)	89 (14.3%)	140 (10.4%)	237 (9.5%)	12 (4.8%)	< 0.001
	No	593 (90.1%)	535 (85.7%)	1200 (89.6%)	2248 (90.5%)	236 (95.2%)	
Long wait times	Yes	86 (13.1%)	139 (22.3%)	216 (16.1%)	265 (10.7%)	15 (6.0%)	< 0.001
	No	572 (86.9%)	485 (77.7%)	1124 (83.9%)	2220 (89.3%)	233 (94.0%)	
Lack of Information	Yes	66 (10.0%)	82 (13.1%)	125 (9.3%)	190 (7.6%)	12 (4.8%)	< 0.001
	No	592 (90.0%)	542 (86.9%)	1215 (90.7%)	2295 (92.4%)	236 (95.2%)	
Language Barriers	Yes	37 (5.6%)	91 (14.6%)	85 (6.3%)	92 (3.7%)	8 (3.2%)	< 0.001
	No	621 (94.4%)	533 (85.4%)	1255 (93.7%)	2393 (96.3%)	240 (96.8%)	
Financial constraints	Yes	32 (4.9%)	53 (8.5%)	62 (4.6%)	93 (3.7%)	4 (1.6%)	< 0.001
	No	626 (95.1%)	571 (91.5%)	1278 (95.4%)	2392 (96.3%)	244 (98.4%)	
Cultural or religious opposition	Yes	26 (4.0%)	29 (4.6%)	37 (2.8%)	73 (2.9%)	5 (2.0%)	0.09
	No	632 (96.0%)	595 (95.4%)	1303 (97.2%)	2412 (97.1%)	243 (98.0%)	
Distance to vaccination centers	Yes	42 (6.4%)	77 (12.3%)	111 (8.3%)	162 (6.5%)	17 (6.9%)	< 0.001
	No	616 (93.6%)	547 (87.7%)	1229 (91.7%)	2323 (93.5%)	231 (93.1%)	
Accessibility issues for people with disabilities	Yes	15 (2.3%)	86 (13.8%)	56 (4.2%)	45 (1.8%)	2 (0.8%)	< 0.001
	No	643 (97.7%)	538 (86.2%)	1284 (95.8%)	2440 (98.2%)	246 (99.2%)	
Other	Yes	26 (4.0%)	15 (2.4%)	17 (1.3%)	22 (0.9%)	5 (2.0%)	< 0.001
	No	632 (96.0%)	609 (97.6%)	1323 (98.7%)	2463 (99.1%)	243 (98.0%)	

* *P*-values were calculated using Chi-square tests to examine the association between reported barriers to vaccination and participants’ education level

Healthcare and government employees reported fewer barriers than self-employed individuals (74.4% vs. 71.1% vs. 56.6%, *p* < 0.001). Difficulty finding centers was significant for self-employed individuals (14.2%) and students (14.0%, *p* < 0.001) (Table 16).

**Table 16:** Distribution of barriers to vaccine by: occupation

Reasons		Healthcare worker	Government Employment	Private sector employee	Self-employed	Unemployed	Retired	Student	Other	Housewife	*p*-value*
Difficulty in finding vaccination centers	Yes	14	76	105	204	49	43	37	8	5	< 0.001
		8.5%	8.4%	8.7%	14.2%	6.7%	11.1%	14.0%	7.6%	5.0%	
	No	150	834	1102	1236	678	343	228	97	95	
		91.5%	91.6%	91.3%	85.8%	93.3%	88.9%	86.0%	92.4%	95.0%	
Long wait times	Yes	10	114	128	239	110	53	46	11	6	< 0.001
		6.1%	12.5%	10.6%	16.6%	15.1%	13.7%	17.4%	10.5%	6.0%	
	No	154	796	1079	1201	617	333	219	94	94	
		93.9%	87.5%	89.4%	83.4%	84.9%	86.3%	82.6%	89.5%	94.0%	
Lack of Information	Yes	12	77	77	145	72	46	30	7	1	< 0.001
		7.3%	8.5%	6.4%	10.1%	9.9%	11.9%	11.3%	6.7%	1.0%	
	No	152	833	1130	1295	655	340	235	98	99	
		92.7%	91.5%	93.6%	89.9%	90.1%	88.1%	88.7%	93.3%	99.0%	
Language Barriers	Yes	5	44	38	140	40	20	20	2	1	< 0.001
		3.0%	4.8%	3.1%	9.7%	5.5%	5.2%	7.5%	1.9%	1.0%	
	No	159	866	1169	1300	687	366	245	103	99	
		97.0%	95.2%	96.9%	90.3%	94.5%	94.8%	92.5%	98.1%	99.0%	
Financial constraints	Yes	8	32	32	83	32	35	18	1	3	< 0.001
		4.9%	3.5%	2.7%	5.8%	4.4%	9.1%	6.8%	1.0%	3.0%	
	No	156	878	1175	1357	695	351	247	104	97	
		95.1%	96.5%	97.3%	94.2%	95.6%	90.9%	93.2%	99.0%	99.0%	
Cultural or religious opposition	Yes	1	28	26	61	27	18	6	1	2	0.02
		0.6%	3.1%	2.2%	4.2%	3.7%	4.7%	2.3%	1.0%	2.0%	
	No	163	882	1181	1379	700	368	259	104	98	
		99.4%	96.9%	97.8%	95.8%	96.3%	95.3%	97.7%	99.0%	98.0%	
Distance to vaccination centers	Yes	8	42	78	165	45	33	28	5	4	< 0.001
		4.9%	4.6%	6.5%	11.5%	6.2%	8.5%	10.6%	4.8%	4.0%	
	No	156	868	1129	1275	682	353	237	100	96	
		95.1%	95.4%	93.5%	88.5%	93.8%	91.5%	89.4%	95.2%	96.0%	
Accessibility issues for people with disabilities	Yes	3	19	13	134	14	12	8	1	0	< 0.001
		1.8%	2.1%	1.1%	9.3%	1.9%	3.1%	3.0%	1.0%	0.0%	
	No	161	891	1194	1306	713	374	257	104	100	
		98.2%	97.9%	98.9%	90.7%	98.1%	96.9%	97.0%	99.0%	100.0%	
Other	Yes	3	8	13	24	11	5	2	8	5	< 0.001
		1.8%	0.9%	1.1%	1.7%	1.5%	1.3%	0.8%	7.6%	5.0%	
	No	161	902	1194	1416	716	381	263	97	95	
		98.2%	99.1%	98.9%	98.3%	98.5%	98.7%	99.2%	92.4%	95.0%	

* *P*-values were calculated using Chi-square tests to assess associations between reported vaccination barriers and participants’ occupational categories

**Fig. 2:**
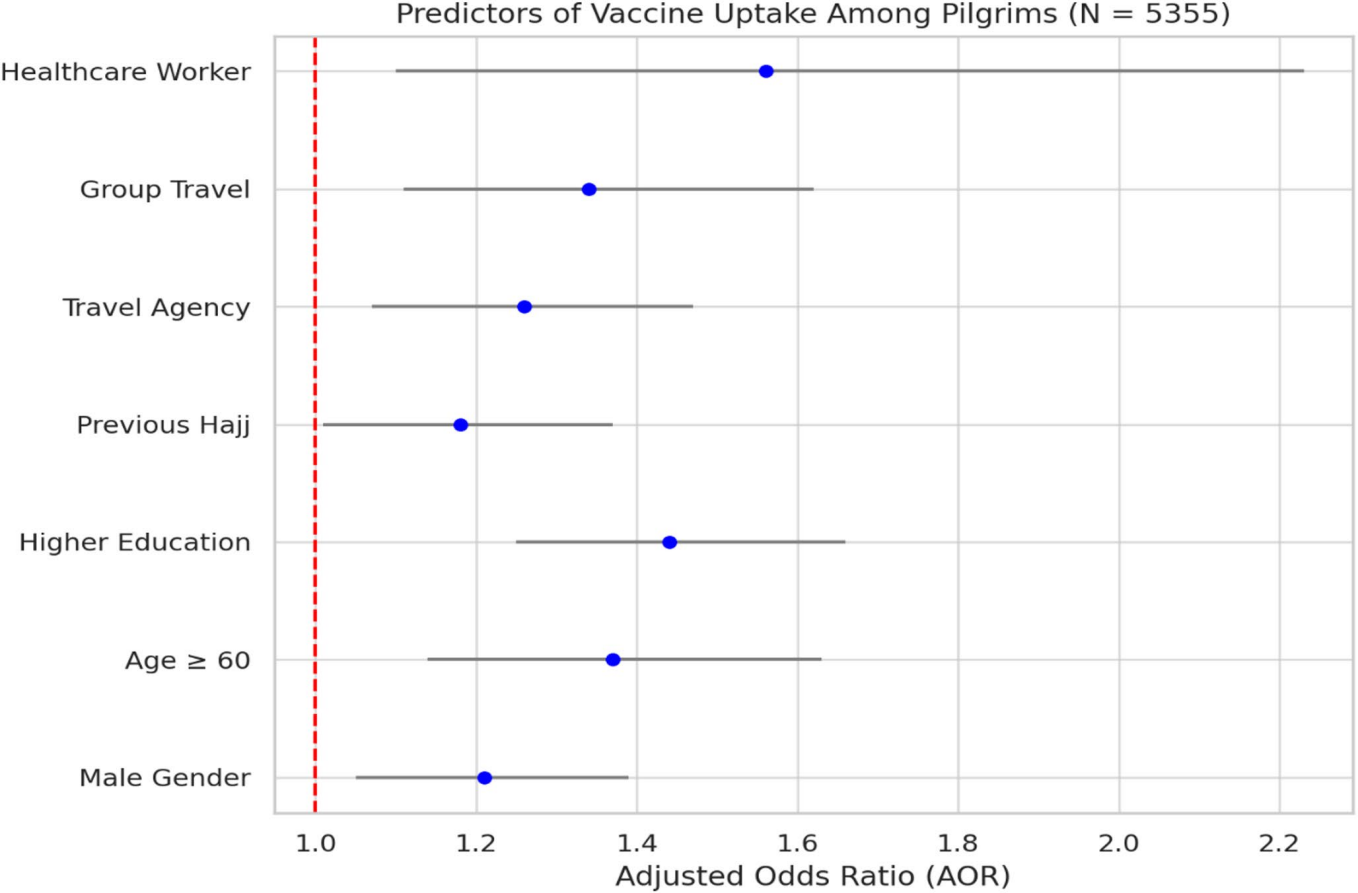
Predictors of vaccine uptake among pilgrims (*N* = 5355)

Figure 2**shows** multivariate logistic regression analysis of predictors associated with vaccine uptake among Hajj and Umrah pilgrims (*N* = 5355). Variables such as being a healthcare worker (AOR = 1.56, 95% CI: 1.10–2.23), higher education (AOR = 1.44, 95% CI: 1.25–1.66), group travel (AOR = 1.34, 95% CI: 1.11–1.62), age ≥ 60 years (AOR = 1.37, 95% CI: 1.14–1.63), and use of a travel agency (AOR = 1.26, 95% CI: 1.07–1.47) were all positively associated with increased likelihood of receiving recommended vaccines. A vertical dashed line at AOR = 1 indicates the null effect.

## Discussion

This study highlights the barriers and successes in vaccinating the Hajj and Umrah pilgrims. The commendable coverage level of 80.3% is attributed to government policies, healthcare providers’ recommendations, and individual reasons. Females’ vaccination rates (83.5%) are slightly higher than male (81.2%), suggesting no need for gender-based targeting. Education and occupation significantly impact acceptance, with higher education and health sector employment leading to greater acceptance. The analysis of vaccine uptake by region revealed significant differences, with the South-East Asia Region showing the highest uptake at 87.4%, followed closely by the Western Pacific Region (86.3%) and the Eastern Mediterranean Region (83.3%). In contrast, the African Region had the lowest uptake at 68.2%, while the Region of the Americas showed a similarly low rate of 49.1%. These differences were statistically significant (*p* < 0.001), thus highlighting the need for regionally tailored strategies to address gaps in vaccine coverage.

While regional variations in vaccine uptake may partly reflect differences in sample size and participant distribution, these factors alone are unlikely to fully explain the disparities observed. For example, African pilgrims were required to show proof of vaccination prior to Hajj visa issuance, yet reported lower uptake possibly due to underreporting or informal verification practices. South-East Asia, despite contributing large pilgrim numbers, was underrepresented in the sample, while the Eastern Mediterranean region was overrepresented and may have faced fewer logistical or regulatory barriers. Beyond sociodemographic factors, differences in health system capacity, access to vaccination, and levels of vaccine hesitancy likely contributed to the regional patterns identified.

While the current vaccination policies seem adequate, some challenges continue to exist. Some key challenges include skepticism about how effective vaccines are, a preference for natural immunity, and challenges around long waiting times and limited services at vaccination centers. These issues are common everywhere, but require more focused approaches, like culture-specific health education and better vaccination infrastructure in areas with low uptake. Instead, existing ones must be optimized. Like other strategies that address peripheral issues, such as increasing access for the self-employed and self-sustaining in poorly performing countries, these should strive to improve coverage levels. At the end, all these findings inform policies so that availability and, as a consequence, health protection during Hajj and Umrah is improved.

The Saudi MoH vaccination mandates, such as meningococcal vaccines, have significantly driven uptake. In our study, 52.1% of vaccinated respondents cited authorities’ requirements as their primary motivator. It had been observed that mandatory vaccination policies reduced meningococcal and influenza outbreaks [5, 7]. Global practices, including the 2016 Summer Olympics, implemented vaccination recommendations against yellow fever and encouraged MMR vaccination to prevent disease transmission [11]. These precautions are consistent with government policies taken during the annual Hajj, which have shown excellent compliance rates to minimize the risks from disease outbreaks [3, 12].

Policy enforcement helps ensure that immunization requirements are met before pilgrims arrive, with compliance verified through pre-travel health checks conducted by authorities. However, this study found inconsistent vaccine coverage among local pilgrims, which may be due to gaps in enforcement or lack of awareness. Strengthening the local health systems and increasing the pre-departure compliance check supervision can close these gaps [13]. Mandating a vaccine certificate (Health Passport) before issuing Hajj permission for domestic pilgrims, like during the COVID-19 pandemic, may be a solution [14].

Vaccinations reduce the severity and transmission of respiratory infections during MGs like Hajj and Umrah, though they do not eliminate risk entirely. Crowded conditions and fatigue facilitate the rapid spread of pathogens, particularly respiratory viruses such as rhinovirus, coronaviruses, and influenza. While influenza vaccination demonstrates 43.4% effectiveness in reducing laboratory-confirmed cases among pilgrims [15]. Suboptimal coverage (26.7% for influenza and 30.5% for pneumococcal vaccines in some cohorts) limits population-level protection [16].

Suboptimal meningococcal vaccination rates among Hajj pilgrims are primarily due to a lack of awareness, limited time to access vaccination services, and occasional cost barriers, particularly among domestic pilgrims. Unlike international pilgrims, who must show proof of vaccination to obtain a visa—many domestic pilgrims are less aware of the requirement, and enforcement is less stringent. addition, non-compliance gaps such as unofficial vaccination documents add to lower coverage.

The non-compliance consequences pose significant risk. The overpopulated conditions that stem from Hajj create an environment that is dangerously conducive to the spread of meningococcal disease as seen in past outbreaks occurring in 1987, 2000, and 2001. Alongside risking personal illness during pilgrimage associated with these outbreaks severely undermines the public’s health by bringing it back to their country. This risk becomes worse due to emerging antibiotic resistance which could add challenges to controlling future outbreaks.

Forging effective compliance will be vital entails tightening controls surrounding education on vaccine prerequisites while serving domestics perks such as free doses makes great strides in ensuring safety against future exposures. Safeguarded fixes are necessary alongside reinforced protocols dealing with vaccination validation safeguards outreach campaigns particularly regarding pre-travel health promotion services.

Precautionary measures such as sanitation through facial masks, hand washing, and limiting crowding reduce the chances of contracting the disease up to 30% [17]. Vaccination combined with other preventive measures proved the best for any disease, covering multiple angles for the best result [18]. Further research must be done on integrated approaches to understand how best to conduct health interventions at MGs [19].

Despite high vaccination rates, this study highlights critical barriers to uptake, including vaccine hesitancy, logistical difficulties, and limited awareness Vaccine hesitancy, driven by beliefs about vaccine inefficacy and reliance on natural immunity, mirrors global vaccine attitudes [20, 21]. Studies have shown that misinformation, cultural beliefs, and fear of side effects contribute to vaccine resistance [22–24]. Addressing these misconceptions requires culturally sensitive health education campaigns involving religious leaders, healthcare providers, and community influencers to build trust and promote evidence-based vaccine information. A Malaysian pilgrim study emphasized the need for improved vaccination infrastructure, including mobile clinics, extended hours, and community-based immunization programs [13]. Strengthening healthcare delivery systems in pilgrims’ home countries is crucial.

A multifaceted approach is crucial to boost vaccine uptake among Hajj pilgrims. First, enhancing health education, confronting infodemics and emphasizing vaccine effectiveness is essential [25]. Second, improved vaccine access through mobile units, extended schedules, and integrated healthcare reduces logistical difficulties [13]. Third, strengthening pre-travel health systems bridges last-minute immunization gaps. Fourth, technological solutions like digital health passports and personalized reminders ensure compliance [26, 27]. Fifth, the disincentives to the reluctant pilgrims include reducing the processing time and the travel fees. Finally, collaboration with WHO, UNICEF, and vaccine manufacturers times influenza vaccine production to match the lunar calendar of Hajj [20]. All these strategies combined can significantly improve vaccination coverage and reduce infectious disease burdens to protect the health of pilgrims.

This study has several limitations that should be acknowledged. First, its cross-sectional design captures data at a single point in time, limiting the ability to draw causal inferences between vaccination uptake and subsequent health outcomes. Second, all data were self-reported, introducing potential recall bias, particularly regarding vaccination history and underlying health conditions. While vaccination status was self-reported by participants, no official documentation, such as immunization cards or visa-related medical records, was requested. Therefore, the responses were based solely on participant recall and awareness of the vaccines received.

No validation of clinical conditions or comorbidities was conducted using medical records. Additionally, the questionnaire did not distinguish between first-time and repeat pilgrims, which may affect interpretation of vaccine awareness and compliance. Selection bias is also possible due to the voluntary nature of participation.

Although data collectors were balanced in gender, the sample consisted of approximately two-thirds male participants. This may reflect the greater accessibility and willingness of male pilgrims to engage in face-to-face interviews in public areas during Hajj and Umrah.

Regarding the separation of Hajj and Umrah data, while we recognize its importance, the study sample included 4794 Hajj pilgrims (89.5%) and only 561 Umrah pilgrims (10.5%). This imbalance limited the feasibility of statistically robust subgroup analyses.

Finally, 130 participants responded “Cannot remember” when asked whether they had recently performed Hajj. These individuals were retained in the dataset to maintain transparency. Their median age was 49 years, and most were from African and Southeast Asian countries. We acknowledge that this group may include individuals with limited literacy, linguistic barriers, or confusion between Hajj and Umrah terminology.

Despite these limitations, this study provides critical insights into vaccination coverage, health risks, and potential gaps in preventive health measures among Hajj and Umrah pilgrims, contributing valuable data to support future public health initiatives. Future studies using longitudinal designs and serological validation are needed for more robust evidence.

## Conclusion

This study illustrates that government policies and healthcare provider recommendations have contributed to increased vaccination uptake among Hajj pilgrims, particularly for mandatory vaccines such as the meningococcal vaccine. However, challenges remain, including vaccine hesitancy, logistical barriers, and occasional mismatches between circulating influenza strains and available vaccines. While the influenza vaccine has been recommended—especially following the H1N1 pandemic, it has not consistently been mandatory, and availability has varied. Expanding the list of recommended vaccines, such as pneumococcal and COVID-19 vaccines, may further support preventive efforts during mass gatherings. A comprehensive strategy involving health education, improved access, and collaboration with international stakeholders is essential. Additionally, promoting complementary preventive measures such as mask-wearing and hand hygiene remains critical to reducing infectious disease transmission. Strengthening health systems, improving awareness, and addressing vaccine inequities will be key to protecting pilgrims’ health in future seasons.

## Data Availability

The data presented in this study are available upon request from the authors.
